# Granulocyte Colony Stimulating Factor Ameliorates Hepatic Steatosis Associated with Improvement of Autophagy in Diabetic Rats

**DOI:** 10.1155/2020/2156829

**Published:** 2020-07-24

**Authors:** Hyun-Woo Joo, Yi-Sun Song, In-Hwa Park, Guang-Yin Shen, Jin-Hee Seong, Na-Kyoung Shin, A-Hyeon Lee, Hyuck Kim, Kyung-Soo Kim

**Affiliations:** ^1^Graduate School of Biomedical Science and Engineering, Hanyang University, Seoul, Republic of Korea; ^2^Department of Internal Medicine, Hanyang University College of Medicine, Seoul, Republic of Korea; ^3^Department of Cardiology, Jilin University Jilin Central Hospital, Jilin, China; ^4^Department of Thoracic and Cardiovascular Surgery, Hanyang University College of Medicine, Seoul, Republic of Korea

## Abstract

**Background:**

We previously reported that the granulocyte colony stimulating factor (G-CSF) ameliorated hepatic steatosis with the enhancement of *β*-oxidation-related gene expression. However, the mechanisms underlying this process remain unclear. This study aimed to determine whether the improvement of hepatic steatosis by G-CSF was associated with autophagy in a rat model of diabetes.

**Methods:**

Eight rats were fed a standard diet, and 16 rats were fed high-fat diet (HFD) for 5 weeks. All HFD-fed rats were then injected with streptozotocin (STZ). One week later, HFD rats injected with STZ were randomly treated with either G-CSF (200 *μ*g/kg/day; diabetes mellitus (DM)/G-CSF) or saline (DM/saline) for 5 consecutive days. Four weeks later, serum biochemical and histology analyses were conducted. The expression of autophagy-associated proteins was determined by Western blotting. The mRNA expression of *β*-oxidation-related genes was determined by quantitative real-time polymerase chain reaction. HepG2 cells were cultured under high glucose (HG) conditions with G-CSF treatment, followed by Oil Red O staining for quantification of lipids.

**Results:**

Histological analysis showed lower lipid accumulation in the DM/G-CSF group than in the DM/saline-treated rats. Protein levels of LC3 and beclin-1 were higher, and those of p62 were lower in the DM/G-CSF rats than in the DM/saline-treated rats. The mRNA expression of *β*-oxidation-related genes was higher in DM/G-CSF rats than in the DM/saline-treated rats. Quantification of lipid levels in HepG2 cells cultured with HG and G-CSF treatment revealed no significant differences.

**Conclusions:**

Our data suggested that G-CSF potentially improves hepatic steatosis and autophagy in the liver of diabetic rats.

## 1. Introduction

Nonalcoholic hepatic steatosis is an initial stage of nonalcoholic fatty liver disease (NAFLD) and is defined as the accumulation of triglycerides (TG) without significant alcohol consumption in the liver [1]. Severe NAFLD can develop and is characterized by nonalcoholic steatohepatitis with increased fibrosis, and it may eventually lead to cirrhosis when combined with other metabolic syndromes [2]. Nonalcoholic hepatic steatosis is an aspect of the metabolic syndrome [3], and it is commonly associated with obesity, insulin resistance, hyperlipidemia, and diabetes [4]. Its worldwide prevalence continues to increase with the growing obesity epidemic and affects approximately 20–40% of adults in developed countries [5].

Autophagy is a process of lysosomal degradation that can degrade intracellular organelles. It can remove damaged cellular components to maintain the homeostasis of cellular energy [6]. Previous evidence has demonstrated that autophagy has a protective function that allows degradation of lipid droplets, while impaired autophagy could contribute to hepatic lipid accumulation [7]. Importantly, recent studies have shown that activation of hepatic autophagy improves nonalcoholic hepatic steatosis as well as alcohol-related liver disease [8].

The granulocyte colony stimulating factor (G-CSF) is a regulator of neutrophil development and formation that is widely used to mobilize bone marrow-derived stem cells in the peripheral blood [9]. G-CSF also improves various liver diseases, including acute liver failure [10], steatohepatitis [11], and liver cirrhosis [12]. Previously, we revealed both the therapeutic and preventive effects of G-CSF on nonalcoholic hepatic steatosis [13, 14]. Although we identified that these effects of G-CSF are related to the reduction in the expression of lipogenesis-related genes and increase in the expression of *β*-oxidation-related genes, the detailed mechanisms underlying the therapeutic effects of G-CSF on hepatic steatosis remain unclear.

In this study, we explored whether the mechanisms of the therapeutic effect of G-CSF were associated with autophagy in a rat model of diabetes. In addition, to identify whether the effect of G-CSF was direct, we investigated the lipid accumulation in HepG2 cells treated with G-CSF.

## 2. Materials and Methods

### 2.1. Animals

This study was performed in accordance with the ARRIVE guidelines on animal research [15]. The Hanyang University Institutional Animal Care and Use Committee approved all experiments. We used 6-week-old male Sprague–Dawley rats (Koatech, Kyungki-do, South Korea). The rats were kept under specific pathogen-free conditions at Hanyang University Medical School Animal Experiment Center and housed in cages under conditions of controlled temperature (23 ± 2°C) and humidity (55 ± 5%) with a 12-hour artificial light and dark cycle.

### 2.2. Animal Model and Experimental Design

**Figure 1:**
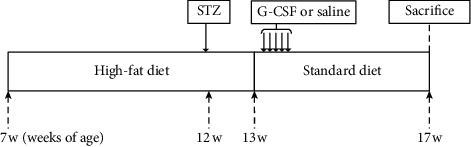
Animal experiment scheme. Sprague–Dawley rats were randomly divided and fed the standard diet (normal control; *n* = 8) or high-fat diet (HFD; *n* = 16) for 6 weeks. After 5 weeks of feeding, HFD rats were intraperitoneally injected with low-dose streptozotocin (STZ; 30 mg/kg). One week later, HFD rats received treatment with either intraperitoneal G-CSF (*n* = 8; 200 *μ*g/kg/day) or saline (*n* = 8; the same volume as G-CSF) for 5 consecutive days and were fed the standard diet until the end of experiment. Four weeks after the start of treatment, all rats were sacrificed for analysis.

The experimental design is shown schematically in Figure 1. Starting at 7 weeks of age, all rats were randomly divided into groups and were given free access to either a standard rodent diet (normal control; *n* = 8) with 5.4% fat content or a high-fat diet (HFD; *n* = 16) with 60% fat content for 6 weeks. After 5 weeks of feeding, all the rats fed with the HFD were injected intraperitoneally with a single low-dose of streptozotocin (STZ; 30 mg/kg) diluted in 0.1 mol/L citrate buffer. Normal control rats were injected with equivalent volumes of citrate buffer. One week later, the HFD-fed rats injected with STZ were randomly selected to receive human recombinant G-CSF (diabetes mellitus (DM)/G-CSF; 200 *μ*g/kg/day; Leucostim, Dong-A Pharmaceutical, Seoul, South Korea; *n* = 8) or an equal volumes of saline (DM/saline; *n* = 8) intraperitoneally for 5 consecutive days. Body weight was determined every week, and blood glucose level was measured at baseline, pretreatment, and posttreatment. The rats were sacrificed under CO_2_ anesthesia for histology and biochemical studies 4 weeks after the beginning of the treatment.

### 2.3. Serum Biochemical Analysis

Blood samples were collected from the tail vein after 8 hours of fasting, and serum was obtained by centrifugation and stored at −70°C. The levels of serum glucose, total cholesterol (TC), TG, aspartate aminotransferase (AST), alanine aminotransferase (ALT), and free-fatty acids (FFAs) were measured by using a model AU400 auto analyzer (Olympus GmbH, Hamburg, Germany) [16]. Insulin resistance (IR) was defined as a homeostasis model assessment of insulin resistance (HOMA-IR) as insulin (*μ*U/mL) × fasting glucose (mmol/L)/22.5 [17].

### 2.4. Histological Analysis

To examine the liver morphology, liver tissues were fixed in 10% formalin solution, embedded in paraffin, and then cut into 3 *μ*m-thick sections for hematoxylin and eosin (H&E) staining. To evaluate the lipid accumulation, frozen 4 *μ*m-thick sections of liver tissues were stained with Oil Red O as previously described [14]. Five fields were randomly selected from the individual slide. The Oil Red O-stained area was analyzed using Image-Pro Plus software (Media Cybernetics, Silver Spring, MD, USA), by calculating the percentage of the ratio of the Oil Red O-positive area to the total tissue area. All histological data were estimated by an independent blinded researcher.

### 2.5. Quantitative Real-Time PCR (q-PCR)

Total RNA was isolated from the liver tissue using QIAzol reagent (Qiagen, Valencia, CA, USA) following the manufacturer's instructions. The RNA (3 *μ*g) was reverse-transcribed with reverse transcriptase (Invitrogen Co., Carlsbad, CA, USA). The mRNA expression levels were quantified using a Light Cycler® 480 System (Roche, Basel, Switzerland) with a FastStart DNA Master SYBR Green I kit (Roche). The genes of interest included those for AMP-activated protein kinase- (AMPK-) 1*α*, AMPK-2*α*, carnitine palmitoyltransferase (CPT)1, and peroxisome proliferator-activated receptor gamma coactivator- (PGC-) 1*α*. The primers used are shown in Table 1. The crossing point of each sample was automatically calculated by the Light Cycler® program. q-PCR analysis for all samples was performed in duplicate. The transcript levels for each gene were determined by normalization against those of glyceraldehyde-3-phosphate dehydrogenase (GAPDH).

### 2.6. Western Blotting

Protein samples were obtained from the liver tissue and homogenized on ice in protein lysis buffer (Pro-prep; iNtRON, Seongnam, South Korea) with a protease inhibitor (Xpert protease inhibitor cocktail solution, GenDEPOT, Barker, TX, USA, 1:100). Forty micrograms of proteins were resolved by 10% SDS-PAGE and transferred onto a polyvinylidene fluoride membrane (Bio-Rad, Hercules, CA, USA). After blocking with 5% bovine serum albumin, the membrane was incubated overnight at 4°C with primary antibodies against LC3, beclin-1, p62, and GAPDH (Cell Signaling Technology, Boston, MA, USA). GAPDH was used as a protein loading control. Protein bands were visualized and quantified using an Image lab 5.0 image analyzer (Bio-Rad).

### 2.7. Cell Culture and Treatment

HepG2 cells were obtained from the American Type Culture Collection (Manassas, VA, USA) and cultured in DMEM (Life Technologies, New York, USA) with normal glucose (5.5 mM) containing 10% FBS (Life Technologies) and 1% streptomycin/penicillin (Life Technologies). Cells were incubated at 37°C in a humidified atmosphere under 5% CO_2_ condition. A cell model for accumulation of intracellular lipids was created by exposure to a high glucose (HG; 45 mM) for 36 hours. For pharmacological studies, HepG2 cells were incubated with either normal glucose (5.5 mM) or HG (45 mM) medium supplemented with indicated concentrations of G-CSF (1, 10, and 100 ng/mL).

### 2.8. Cell Viability

HepG2 cells were cultured overnight, and then incubated with increasing concentrations (1, 10, 100, and 1000 ng/mL) of G-CSF for 36 hours. Subsequently, cells were treated with methylthiazolyldiphenyl-tetrazolium bromide (MTT) solution (0.5 mg/mL) diluted in the medium and incubated for an additional another 2 hours. The culture medium was removed and the intracellular formazan product was dissolved in dimethylsulfoxide (DMSO). The absorbance was measured at a wavelength of 595 nm using an iMark™ Microplate Absorbance Reader (Bio-Rad). MTT assays were performed in triplicate.

### 2.9. Quantification of Lipids by Oil-Red O Staining

To prepare Oil Red O solution, 0.35 g Oil Red O (Sigma-Aldrich, St. Louis, MO, USA) per 100 mL of 100% isopropanol was mixed to yield an isopropanol concentration of 60%; this solution was then filtered. Briefly, the HepG2 cells were washed with PBS, followed by fixation with 10% formalin for 30 minutes. After washing with distilled water, fixed cells were washed with 60% isopropanol shortly and then stained with Oil Red O solution for 30 minutes at room temperature. Subsequently, after washing with distilled water, the Oil Red O was eluted with 100% isopropanol for 15 minutes at room temperature, and it is quantified by measuring the absorbance at a wavelength of 490 nm using an iMark™ Microplate Absorbance Reader (Bio-Rad).

### 2.10. Statistical Analysis

All statistical analyses were conducted using SPSS 21.0 for Windows (IBM, Armonk, NY, USA). The results are presented as the means ± standard deviations, except for the results of the histological analyses, which are presented as the means ± standard errors of the means. Comparisons of parameters among groups were performed using one-way analysis of variance (ANOVA) followed by a post hoc Tukey's test. *P* values <0.05 were considered statistically significant.

## 3. Results

### 3.1. Body Weight and Serum Biochemical Analysis

**Figure 2:**
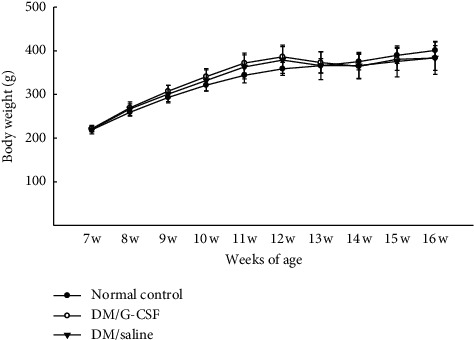
Changes in body weight. Alterations of body weight in Sprague–Dawley rats were monitored during the entire experimental period. DM, diabetes mellitus. All data are expressed as mean ± standard deviation (*n* = 8 per group).

The body weights of the rats from each group were not significantly different (Figure 2). At the end of the experiment, circulating glucose levels were significantly higher in the HFD-fed rats injected with STZ than in the rats form the normal control group but were similar between the DM/G-CSF and DM/saline groups (ANOVA: *F*_(2,9)_ = 15.682, *P* < 0.05). HOMA-IR was significantly lower in the DM/G-CSF group than in the DM/saline group and did not differ between the normal control group and the DM/G-CSF group (ANOVA: *F*_(2,9)_ = 13.494, *P* < 0.05). Finally, levels of circulating TG, AST, ALT, and FFAs were similar for all groups. TC levels were significantly higher in the DM/saline group than in the normal control group (Table 2).

### 3.2. Hepatic Lipid Accumulation

**Figure 3:**
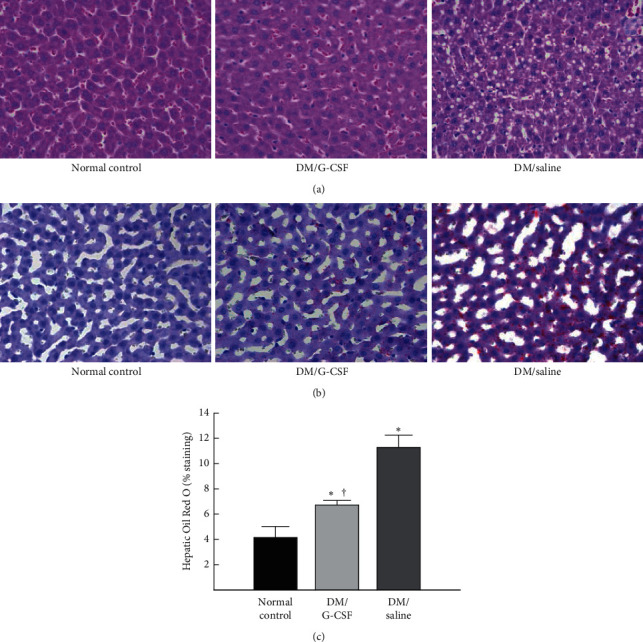
Histological changes in the liver. (a) Representative images of hematoxylin and eosin (H&E) staining in the liver sections from each treatment group (magnification ×400). (b) Liver sections were stained with Oil Red O (magnification ×400). (c) Quantitative analysis was conducted in Oil Red O-stained sections, and the mean area (%) occupied by Oil Red O-positive area was calculated for five randomly selected fields of each tissue section. DM, diabetes mellitus. All data are expressed as the means ± standard errors (*n* = 8 per group). ^*∗*^*P* < 0.05 vs. normal control. ^†^*P* < 0.05 vs. DM/saline.

The progression of hepatic steatosis was verified by H&E and Oil Red O staining. At the end of the experiment, the liver tissues from the DM/saline group showed severe micro- and macrovesicular steatosis and numerous ballooning hepatocytes. In contrast, the liver tissues from the DM/G-CSF group exhibited no fat accumulation; this was not different from the case for the liver tissues of rats from the normal control group (Figure 3(a)). The accumulation of lipid droplets as indicated by Oil Red O staining was significantly lower in the DM/G-CSF group than in the DM/saline group and was significantly different between the DM/G-CSF group and normal control group (Figures 3(b) and 3(c)) (ANOVA: *F*_(2,82)_ = 79.387, *P* < 0.05).

### 3.3. Expression of Proteins Related to Autophagy

**Figure 4:**
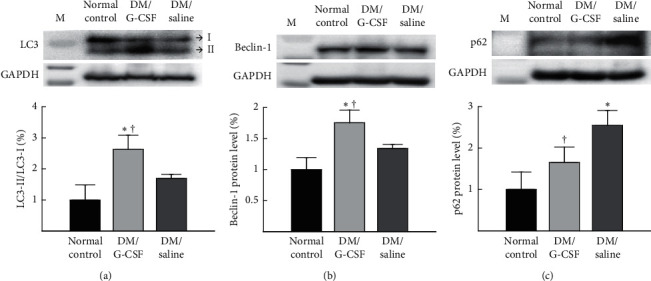
Expression of proteins related to autophagy in the liver. The levels of microtubule-associated protein light chain 3 (LC3) (a), beclin-1 (b), and p62 (c) proteins in the liver tissues were measured by Western blotting. The representative images of the Western blotting analyses are displayed on the upper panels, and quantitative densitometry analysis is shown in the lower panels. LC3 expression was normalized by calculating LC3 conversion as the ratio of LC3-II/LC3-I expression. Beclin-1 and p62 expression levels were normalized by comparison with the levels of glyceraldehyde-3-phosphate dehydrogenase (GAPDH). M, marker and DM, diabetes mellitus. All data are expressed as the means ± standard deviations (*n* = 8 per group). ^*∗*^*P* < 0.05 vs. normal control. ^†^*P* < 0.05 vs. DM/saline.

To confirm whether the ameliorative effects of G-CSF on hepatic steatosis were associated with autophagy, we examined the protein expression of autophagy markers, including LC3, beclin-1, and p62 by Western blotting. The protein level of the LC3, as determined by calculating the LC3 conversion (LC3-II/LC3-I), was significantly higher in the DM/G-CSF group than in the DM/saline group and the normal control group (Figure 4(a)) (ANOVA: *F*_(2,8)_ = 15.552, *P* < 0.05). The protein level of beclin-1 was significantly higher in the DM/G-CSF group than in the DM/saline group and the normal control group (Figure 4(b)) (ANOVA: *F*_(2,7)_ = 16.446, *P* < 0.05). The protein level of p62 was significantly lower in the DM/G-CSF group than in the DM/saline group, and it was not significantly different from that in the normal control group (Figure 4(c)) (ANOVA: *F*_(2,10)_ = 16.457, *P* < 0.05).

### 3.4. Expression of mRNAs Related to *β*-oxidation

**Figure 5:**
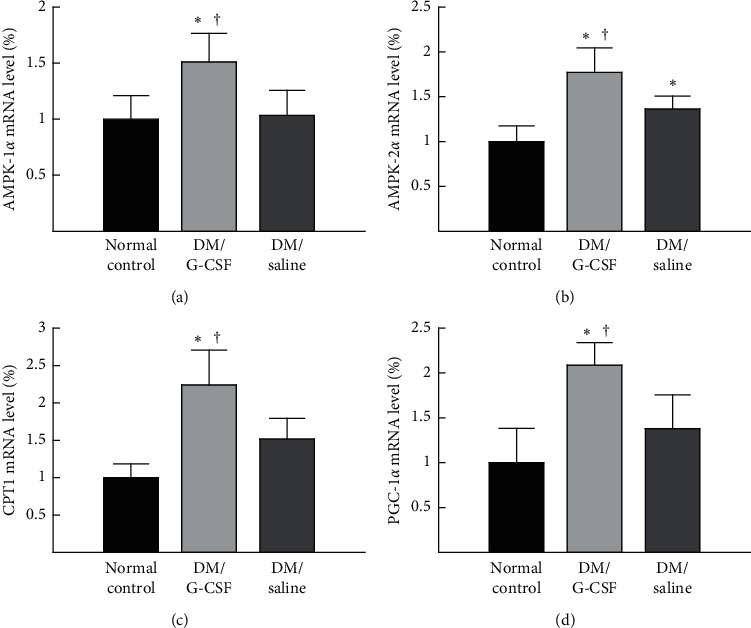
mRNA expression levels of *β*-oxidation-related genes in the rat liver. mRNA expression of AMP-activated protein kinase (AMPK)-1*α* (a), AMPK-2*α* (b), carnitine palmitoyltransferase (CPT)1 (c), and the peroxisome proliferator-activated receptor gamma coactivator (PGC)-1*α* (d) was measured by quantitative real-time polymerase chain reaction (q-PCR) in duplicate. The transcript levels were normalized by comparison with glyceraldehyde-3-phosphate dehydrogenase (GAPDH) expression. DM, diabetes mellitus. All data are expressed as the means ± standard deviations (*n* = 8 per group). ^*∗*^*P* < 0.05 vs. normal control. ^†^*P* < 0.05 vs. DM/saline.

To investigate the alteration of the expression of *β*-oxidation-related genes by G-CSF, mRNA levels of to *β*-oxidation-related genes, including AMPK-1*α*, AMPK-2*α*, CPT1, and PGC-1*α*, in the liver were examined by q-PCR. The mRNA level of AMPK-1*α* was significantly higher in the DM/G-CSF group than in the DM/saline group and the normal control group (Figure 5(a)) (ANOVA: *F*_(2,13)_ = 8.615, *P* < 0.05). The mRNA level of AMPK-2*α* was significantly higher in the DM/G-CSF group than in the DM/saline group and the normal control group; there was a significant difference between the mRNA level of AMPK-2*α* in the DM/saline group and the normal control group (Figure 5(b)) (ANOVA: *F*_(2,14)_ = 20.619, *P* < 0.05). The mRNA level of CPT1 was significantly higher in the DM/G-CSF group than in the DM/saline group and the normal control group (Figure 5(c)) (ANOVA: *F*_(2, 11)_ = 17.477, *P* < 0.05). The mRNA level of PGC-1*α* was significantly higher in the DM/G-CSF group than in the DM/saline group and the normal control group (Figure 5(d)) (ANOVA: *F*_(2,11)_ = 11.673, *P* < 0.05).

### 3.5. Effect of G-CSF on Lipid Accumulation in HepG2 Cells

**Figure 6:**
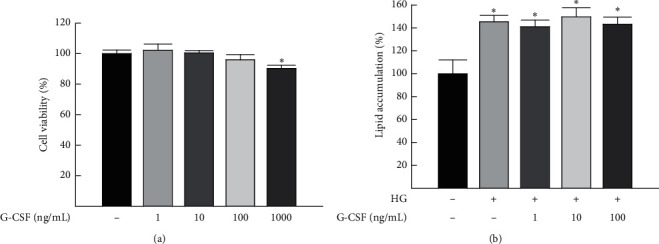
Effect of G-CSF on the viability and high glucose- (HG-) induced lipid accumulation in HepG2 cells. (a) Viability of HepG2 cells after treatment with increasing doses of G-CSF for 36 hours was measured by the MTT assay in triplicate. (b) Lipid accumulation was assessed by quantification of Oil Red O staining in HepG2 cells, which were cultured with either 5.5 mM or 45 mM HG medium, followed by cotreatment with increasing concentrations of G-CSF for 36 hours. All data are expressed as the means ± standard deviations (*n* = 3 per group). ^*∗*^*P* < 0.05 vs. no treatment.

Various concentrations of G-CSF (1, 10, 100, and 1000 ng/mL) were added to the culture medium of HepG2 cells. After 36 hours, cell viability was measured using the MTT assay. Treatment with G-CSF at concentrations up to 100 ng/mL had no cytotoxic effects on HepG2 cells. However, treatment with G-CSF at a concentration of 1000 ng/mL reduced cell viability, as observed by comparisons with the untreated group (Figure 6(a)). To determine whether G-CSF is directly involved in HG-induced lipid accumulation, HepG2 cells were treated with noncytotoxic concentrations of G-CSF (1, 10, and 100 ng/mL) in the presence of HG conditions for 36 hours, and lipid accumulation was quantified by Oil Red O staining. Intracellular lipid accumulation was significantly higher in the HepG2 cells exposed to HG conditions than in the HepG2 cells exposed to normal glucose conditions. However, cotreatment with HG and increasing amounts of G-CSF (1, 10, and 100 ng/mL) had no effect on lipid accumulation in the HepG2 cells (Figure 6(b)).

## 4. Discussion

In the present study, we examined the effects of G-CSF treatment on hepatic steatosis in a rat model. We demonstrated that G-CSF treatment emeliorated *β*-oxidation as well as hepatic steatosis in the liver tissue. Moreover, G-CSF induced autophagy activation, which provides a molecular explanation for the therapeutic effects of G-CSF on hepatic steatosis. We also confirmed that G-CSF did not affect the lipid accumulation in the HepG2 liver cancer cells, which indicates that G-CSF may not have a direct beneficial effect on hepatic steatosis by signaling through the G-CSF receptor.

Growing evidence has demonstrated the contribution of autophagy in the regulation of lipid metabolism. There have been studies that activation of autophagy could reduce the number of lipid droplets resulting in therapeutic effects against NAFLD [18], while defective autophagy might trigger the accumulation of lipids leading to the development of NAFLD [19]. In the present study, autophagy was activated in the liver of rats treated with G-CSF, with higher levels of beclin-1 protein and LC3-II/LC3-I (LC3 conversion), and lower levels of expression of p62 protein in rats treated with G-CSF. Beclin-1 is a major indicator of the autophagic activity; it forms a kinase complex with class III phosphatidylinositol 3-kinase [20]. LC3 is a representative marker of the activation of autophagy. It is located specifically on the outer membrane of autophagosomes, which forms the phagophore and induces lysosomal degradation [21]. The expression of LC3 could be reported as LC3 conversion because LC3-I is conjugated to phosphatidylethanolamine to form LC3-II, which is essential for the formation of the autophagosome when autophagy is initiated [22]. P62 links LC3 and ubiquitinated substrates for delivery to the autophagosome, which is efficiently degraded by lysosomal acid hydrolases during the normal autophagic activity [23]. Thus, it has been suggested that increased p62 levels relate to suppressed autophagy, and decreased p62 levels relate to autophagy activation [24]. A recent study revealed that autophagy activation following treatment with a polyphenol fraction could prevent nonalcoholic hepatic steatosis by upregulation of autophagy markers, such as beclin-1, and LC3 conversion with decreased p62 levels in the liver tissue of rats fed a cafeteria diet [25]. Therefore, with regard to the results of our study, G-CSF treatment may decrease lipid droplet formation by enhancing autophagy, which may be considered a beneficial effect of G-CSF treatment on hepatic steatosis.

Autophagy contributes to the generation of fatty acids by breaking down lipid droplets and formation of the autolysosome, which is an autophagosome combined with a lysosome [7, 26]. The fatty acids generated from the lipid droplets by autophagy are consumed by mitochondrial *β*-oxidation, a process that is critical for the disposal of fatty acids [27, 28]. *β*-oxidation has an important role on the development of fatty liver disease and nonalcoholic steatohepatitis [29]. In conditions of excessive influx of lipids in hepatocytes, defective *β*-oxidation can lead to the accumulation of fatty acids, which causes NAFLD [30]. Furthermore, unless fatty acids generated from activated autophagy are removed quickly, they may also be harmful to hepatocytes due to their toxicity [31]. Thus, concurrent increases in *β*-oxidation and the autophagy activity would be ideal targets for NAFLD therapy. Presently, G-CSF increased the expression of *β*-oxidation genes, such as AMPK-1*α*, AMPK-2*α*, CPT1, and PGC-1*α*, accompanied with the enhancement of autophagy in the rat liver. Further studies are needed to determine whether increased *β*-oxidation is dependent on G-CSF-induced autophagy, and whether protein expression levels of these genes are similar to the corresponding mRNA expression.

NAFLD is a common chronic liver disease and is closely associated with type 2 diabetes mellitus (T2DM) [32]. NAFLD and T2DM are components of metabolic syndrome and share a number of related clinical features such as IR, dyslipidemia, and visceral obesity [33]. NAFLD has an estimated prevalence of approximately 70% in patients with T2DM [34]. In our study, a rat model of NAFLD was developed using HFD feeding and low-dose STZ injection. The model mimicked the metabolic characteristics and a natural history of T2DM in humans [35]. We identified hepatic steatosis via the histological evaluation of the liver tissues from our rat model, indicating that these rats with T2DM also had NAFLD. In addition, we showed that G-CSF treatment reduced hepatic steatosis in the rats, suggesting the potential of G-CSF for the treatment of hepatic steatosis in T2DM.

Previous studies have suggested several potential mechanisms underlying the therapeutic effect of G-CSF on various liver diseases, including the induction of bone marrow-derived cell mobilization and direct effects of the G-CSF receptor-mediated signaling pathway [12, 36]. Our previous study also confirmed the presence of the G-CSF receptor in the rat liver tissue [14]. However, G-CSF treatment had no effect on lipid accumulation in HepG2 cells. Taken together, our data support the idea that the therapeutic effects of G-CSF might be associated with the induction of bone marrow-derived cell mobilization by G-CSF rather than a direct effect resulting from G-CSF receptor activation in the liver.

This study has several limitations. First, we were unable to investigate the therapeutic mechanisms underlying the action of G-CSF on hepatic steatosis, in association with its effects on bone marrow-derived mobilization by G-CSF. This will require further investigations of the bone marrow-dependent effects of G-CSF. Second, treatment regimens including dosage, timing, and duration of G-CSF need to be established. Finally, our study did not analyze the expression levels of other autophagy markers or histological evidence related to autophagy. Additional studies are needed to more precisely evaluate the mechanisms involved in autophagy.

In conclusion, our study showed that G-CSF improved hepatic steatosis and induced the activation of *β*-oxidation and autophagy in a rat model. We speculate that the activation of autophagy by G-CSF could be associated with the reduction in hepatic steatosis through the upregulation of *β*-oxidation-related genes. In addition, we found that G-CSF did not reduce intracellular lipid accumulation, which implies that the therapeutic effect of G-CSF may be related to bone marrow mobilization.

## Figures and Tables

**Table 1:** Primer sequences used in this study.

Primer	Sequence	Size (bp)
AMPK-1*α*	F: 5′-CAA-AGA-TTT-CTA-CTT-GGC-AAC-AAG-C-3′ R: 5′-CAA-CCA-AGA-ATG-GTA-CTC-TCT-CAG-G-3′	95

AMPK-2*α*	F: 5′-CGC-ACT-GTG-AAA-TCA-CAT-ACA-CTC-T-3′ R: 5′-GTC-ACC-TGG-GAA-TGT-TCA-CAT-AAC-T-3′	95

CPT1	F: 5′-GCT-TAT-CTT-CTA-ATC-CCA-CCC-AGT-C-3′ R: 5′-CCA-TTA-CTT-GAG-ATC-CCT-GAG-TCT-G-3′	99

PGC-1*α*	F: 5′-CCT-GAA-CTT-GAC-CTT-TCT-GAA-CTT-G-3′ R: 5′-ATT-GGT-CAC-TAC-ACC-ACT-TCA-ATC-C-3′	85

GAPDH	F: 5′-CCT-TCT-CTT-GTG-ACA-AAG-TGG-ACA-T-3′ R: 5′-CGT-GGG-TAG-AGT-CAT-ACT-GGA-ACA-T-3′	96

AMPK-1*α*, AMP-activated protein kinase-1*α*; AMPK-2*α*, AMP-activated protein kinase-2*α*; CPT1, carnitine palmitoyltransferase 1; PGC-1*α*, peroxisome proliferator-activated receptor gamma coactivator-1*α*; GAPDH, glyceraldehyde-3-phosphate dehydrogenase; F, forward; and R, reverse.

**Table 2:** Results of serum biochemical analysis 4 weeks after the start of G-CSF or saline treatment.

	Normal control	DM/G-CSF	DM/saline
Glucose (mg/dL)	130.33 ± 6.66	288.50 ± 67.00^*∗*^	323.20 ± 43.42^*∗*^
TC (mg/dL)	90.88 ± 5.14	102.08 ± 7.55	109.59 ± 16.49^*∗*^
TG (mg/dL)	27.31 ± 4.00	29.60 ± 8.85	44.34 ± 27.87
AST (U/L)	163.94 ± 19.16	174.04 ± 35.49	193.43 ± 28.30
ALT (U/L)	78.48 ± 10.87	76.50 ± 22.53	83.08 ± 36.37
FFAs (*μ*Eq/L)	591.13 ± 136.74	656.83 ± 105.40	754.25 ± 294.55
HOMA-IR	1.65 ± 0.28	2.40 ± 0.19^†^	3.44 ± 0.50^*∗*^

TC, total cholesterol; TG, triglyceride; AST, aspartate aminotransferase; ALT, alanine aminotransferase; FFAs, free fatty acids; HOMA-IR, homeostasis model assessment of insulin resistance; and HOMA-IR = fasting insulin (*μ*U/mL) × fasting glucose (mmoL/L)/22.5. Normal control, standard diet with no treatment; DM/G-CSF, high-fat diet (HFD) and low-dose streptozotocin (STZ) injection with G-CSF treatment; and DM/saline, HFD and low-dose STZ injection with saline treatment. All data are expressed as the means ± standard deviations. ^*∗*^*P* < 0.05 vs. normal control. ^†^*P* < 0.05 vs. DM/saline.

## Data Availability

The data used to support the findings of this study are available from the corresponding author upon request.
